# On the molecular identity of *Paratylenchus nanus* Cobb, 1923 (Nematoda: Tylenchida)

**DOI:** 10.21307/jofnem-2020-127

**Published:** 2021-01-11

**Authors:** Sergei A. Subbotin, Guiping Yan, Mihail Kantor, Zafar Handoo

**Affiliations:** 1 Plant Pest Diagnostic Center, California Department of Food and Agriculture, 3294 Meadowview Road, Sacramento, CA 95832; 2 Center of Parasitology of A.N. Severtsov Institute of Ecology and Evolution of the Russian Academy of Sciences, Leninskii Prospect 33, Moscow 117071, Russia; 3 Department of Plant Pathology, North Dakota State University, Fargo, ND 58108; 4 Mycology and Nematology Genetic Diversity and Biology Laboratory, USDA, ARS, Building 010 A, BARC-West, 10300 Baltimore Avenue, Beltsville, MD 20705

**Keywords:** COI, D2-D3 of 28S rRNA, ITS rRNA, Molecular phylogeny, P. nanus type A, P. nanus type B, Paratylenchus projectus

## Abstract

In this study, molecular characterization of *Paratylenchus nanus* collected from the type locality in Four Mile Run, Fall Church, Virginia using *COI,* D2-D3 of 28 S rRNA and ITS rRNA gene sequences was provided. We molecularly also characterized, *Paratylenchus* specimens collected from grasses in Devils Lake, Ramsey County, North Dakota indicated as the type locality in the original description of *P. nanus* by Cobb (1923). These nematodes were identified as representatives of the species *P. projectus.* Populations of *P. nanus* belonging to the molecular types A and B, and previously designated by Van den Berg et al. (2014) should be now identified as *P. nanus* and *P. projectus*, respectively.

*Paratylenchus nanus* was described by Cobb (1923) from female specimens collected from soil near the roots of grasses, Devils Lake, North Dakota, in April 1915 and later from Four Mile Run, Fall Church, Virginia, in August 1922. Some of Cobb’s original notes were available to Tarjan (1960) including formula measurements of a single female from North Dakota and three females from Virginia. Tarjan (1960) also found Cobb’s type specimens for study including a single female from North Dakota and one female plus the anterior part of another from Virginia. He selected the specimen from Virginia as lectotype of *P. nanus* (Raski, 1975). Geraert (1965) in his review of the genus *Paratylenchus*, suggested that *P. nanus* should be a synonym of *P. bukowinensis.* Similarity and possible synonymization with *P. bukowinensis* have been already mentioned by Cobb (1923) in the *P. nanus* species description. Later, Thorne and Smolik (1971) re-described and illustrated *P. nanus* from specimens collected from native sod near Devils Lake, North Dakota and designated the specimens as topotypes. However, Raski (1975) believed that the species from Devils Lake collected by Thorne and Smolik (1971) belonged to *P. projectus* Jenkins 1956, not to *P. nanus*, and designated new topotypes collected in 1958 from a grass soil sample in Fall Church, Virginia. Raski (1975) provided a detailed report of various collections and descriptions of the species. He also noticed that *P. nanus* was very similar to *P. projectus.*

Van den Berg et al. (2014) provided a detailed molecular and morphological characterization of several populations identified as *P. nanus* from South Africa and California, USA. This study showed that the populations identified as *P. nanus* might indeed contain two sibling species (molecular types A and B), which were well separated using molecular criteria, but had very similar morphometrics. These authors also noticed intraspecific variation in shapes of lip region and tail. Thus, the designation of the true *P. nanus* species remained to be unresolved.

The objective of this work was to molecularly characterize *P. nanus* from the type locality, Four Mile Run, Fall Church, Virginia, USA designated by Tarjan (1960) and *Paratylenchus* species from the roots of grasses collected in Devils Lake, North Dakota, which was also indicated as the type locality in the original description of *P. nanus* by Cobb (1923).

## Materials and methods

### Nematode samples

Several sampling trips were conducted to Devils Lake, Ramsey County, North Dakota and Four Mile Run, Fall Church, Virginia to collect nematodes. Soil samples were arbitrarily collected from the grassland in Devils Lake, Ramsey County, North Dakota in 2015 and 2016 (coordinates: 48.10805 N, 98.94384 W). Sampling was conducted from June to October each year. In each sampling spot, the top, dry soil (1–2 cm) was removed and the remaining soil was collected up to a depth of 30 cm using a soil probe (2.5 cm in diameter and 30 cm in depth). Each soil sample consisted of a composite of 10 to 15 soil cores and soil samples were placed in plastic bags. The soil with grasses was also placed in a glasshouse for several weeks and then sampled for nematodes (nematode sample codes = CD1902, CD1902, CD1904, CD2192 and CD2403). Several soil samples were also collected along Four Mile Run, Fall Church, Virginia in September 2020. Nematodes were extracted from soil using sieving and decanting as well as the sugar centrifugal-flotation method (Jenkins, 1964). One sample (N 4, coordinates: 38.52563 N, 77,08557 W) contained *Paratylenchus* nematodes (nematode sample code = CD3326)

### Morphological examination

Several *Paratylenchus* female specimens extracted from grass soil samples were morphologically examined and photographed with an automatic Infinity 2 camera attached to a compound Olympus BX51 microscope equipped with a Nomarski interference contrast, and then these specimens were used for molecular study.

### DNA extraction, PCR, sequencing and phylogenetic analysis

DNA was extracted from several specimens using the proteinase K protocol. DNA extraction and PCR protocols were as described by Tanha Maafi et al. (2003). The primer sets D2A (5’ – ACA AGT ACC GTG AGG GAA AGT TG – 3’) and D3B (5’ – TCG GAA GGA ACC AGC TAC TA – 3’) amplifying the D2-D3 of 28 S rRNA gene (Subbotin et al., 2006), TW81 (5’ – GTT TCC GTA GGT GAA CCT GC – 3’) and AB28 (5’ – ATA TGC TTA AGT TCA GCG GGT – 3’) amplifying ITS rRNA (Tanha Maafi et al., 2003) and COIF5 (5’ – AAT WTW GGT GTT GGA ACT TCT TGA AC – 3’) and COIR9 (5’–CTT AAA ACA TAA TGR AAA TGW GCW ACW ACA TAA TAA GTA TC – 3’) amplifying the partial *COI* gene (Powers et al., 2014) were used in this study. PCR products were purified using the QIAquick Gel Extraction Kit (Qiagen) according to the manufacturer’s instructions and submitted to direct sequencing at GENEWIZ (CA, USA). The new *Paratylenchus* sequences were submitted to the GenBank database under accession numbers: MT668705, MT668708-MT668712, MW234449, MW234450, MW234452, MW238473-MW238475.

The new sequences for each gene (D2-D3 of 28 S rRNA, ITS rRNA, *COI*) were aligned using ClustalX 1.83 (Thompson et al., 1997) with their corresponding published gene sequences (Subbotin et al., 2006; Van den Berg et al., 2014; Munawar et al., 2018; Etongwe et al., 2020; Mwamula et al., 2020; Powers et al., 2020 and others). ClustalX with default parameters (gap opening = 15 and gap extension = 6.66) was used to generate the ITS rRNA and *COI* gene sequence alignments, whereas the modified parameters (gap opening = 5 and gap extension = 3) were applied to generate the D2–D3 of 28 S rRNA gene alignment. Sequence datasets were analyzed with Bayesian inference (BI) using MrBayes 3.1.2 as described by Van den Berg et al. (2014).

## Results and discussion

### Morphological characterization of *Paratylenchus nanus* and *P. projectus*

Several *Paratylenchus* female specimens extracted from a sample collected in Four Mile Run, Fall Church, Virginia were morphologically similar to those described by Cobb (1923), Raski (1975) and Van den Berg et al. (2014) and identified here as *P. nanus*. Measurements of eight females were: L = 377.1 ± 13.9 (356.0–401.3) µm; W = 17.0 ± 0.9 (16.0–18.9) µm; a = 22.2 ± 1.1 (20.6–23.5); b = 3.7 ± 0.2 (3.4–3.9); c = 16.0 ± 2.3 (13.7–18.4); V = 83.0 ± 1.6 (80.9–85.0)%; stylet = 31.2 ± 0.8 (30.3–32.4) µm; pharynx = 101.2 ± 2.9 (96.0–104.3) µm; anterior end to median bulb = 56.9 ± 1.8 (53.8–60.0) µm; anterior end to excretory pore = 78.3 ± 3.2 (73.8–83.3) µm; tail = 23.1 ± 3.4 (20.2–26.9) µm.

Two females and several juveniles extracted from samples collected from grasses in Devils Lake, Ramsey County, North Dakota were similar to those described by Raski (1975) and Van den Berg et al. (2014) and identified here as *P. projectus*. Females had short (330 µm) body and stylet 28–31 µm long.

**Figure 1:**
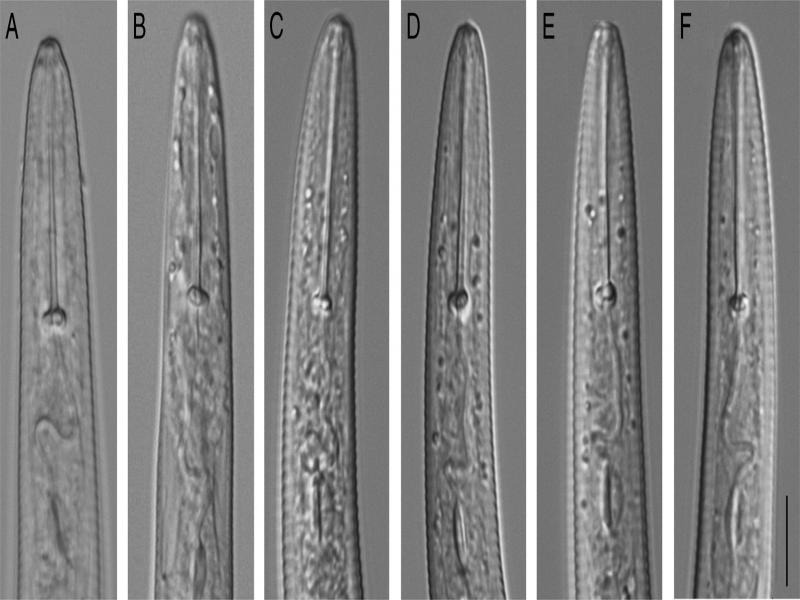
Anterior regions of *Paratylenchus.* A, B: *P. projectus* from samples collected in Devils Lake, Ramsey County, North Dakota; C-F: *P. nanus* from samples collected in Four Mile Run, Fall Church, Virginia. Scale – 10 µm.

**Figure 2:**
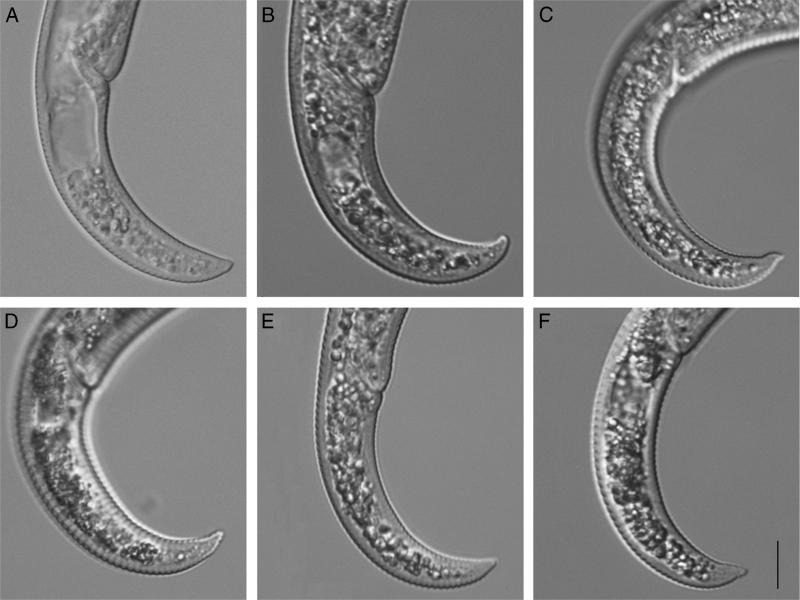
Posterior regions of *Paratylenchus.* A: *P. projectus* from samples collected in Devils Lake, Ramsey County, North Dakota; B-F: *P. nanus* from samples collected in Four Mile Run, Fall Church, Virginia. Scale – 10 µm.

Morphological examination of specimens showed that *P. nanus* is very similar to *P. projectus* and the characters most important in distinguishing these two species as indicated by Raski (1975) are: (i) lip region which is truncate, often set off by slight but distinct narrowing, annuli indistinct in *P. projectus,* head rounded with distinct annuli, not set off, in *P. nanus* (Figure 1) and (ii) tail bluntly rounded, often digitate in *P. projectus;* subacute in *P. nanus* (Figure 2).

### Molecular characterization and relationships

#### The D2-D3 of 28 S rRNA gene

**Figure 3:**
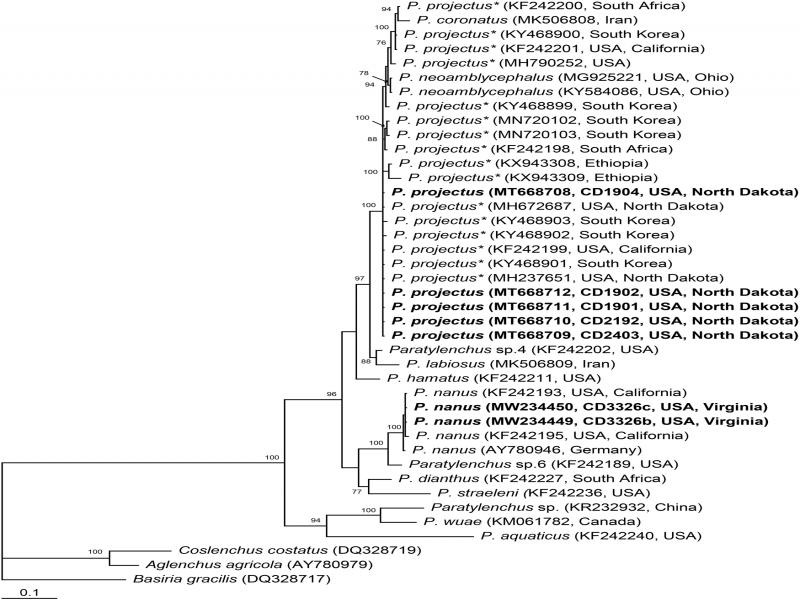
Phylogenetic relationships of *Paratylenchus nanus* with other related species. Bayesian 50% majority rule consensus tree from two runs as inferred from analysis of the D2-D3 of 28 S rRNA gene sequence alignment under the GTR + I + G model. Posterior probabilities equal or more than 70% are given for appropriate clades. New sequences are indicated in bold. * – originally identified as *P. nanus.*

The alignment generated with modified parameters was 756 bp in length and contained 41 sequences. Phylogenetic relationships of *P. nanus* within selected *Paratylenchus* are given in Figure 3. Sequences of *P. nanus* from Virginia were identical to that of *P. nanus* type A from California provided by Van den Berg et al. (2014). Intraspecific variation for this species was up to 0.5%. New sequences of *P. projectus* from North Dakota were identical to those previously identified as *P. nanus* type B from California (Van den Berg et al., 2014) or as *P. nanus* from North Dakota (Upadhaya et al., 2019a, b) and South Korea (Mwamula et al., 2020). All these sequences are now considered as representatives of *P. projectus.*

### The ITS of rRNA gene

**Figure 4:**
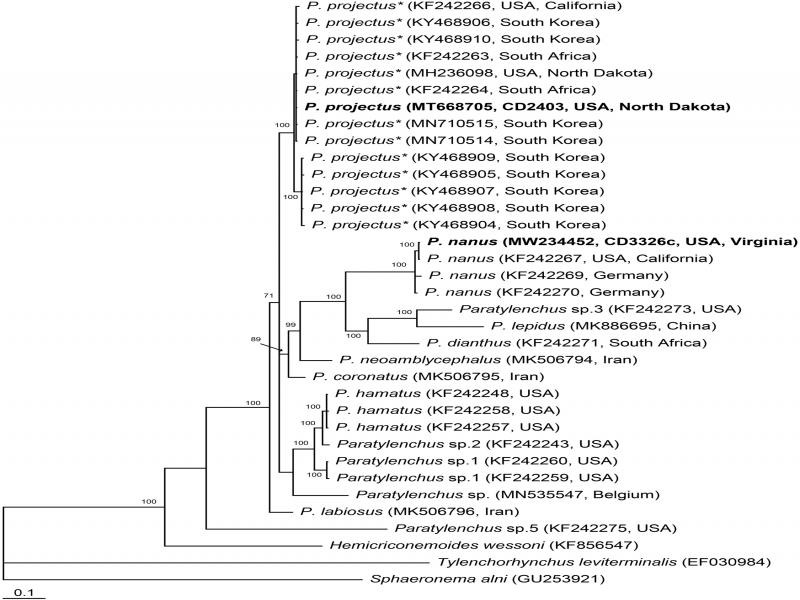
Phylogenetic relationships of *Paratylenchus nanus* with other related species: Bayesian 50% majority rule consensus tree from two runs as inferred from analysis of the ITS rRNA gene sequence alignment under the GTR + I + G model. Posterior probabilities equal or more than 70% are given for appropriate clades. New sequences are indicated in bold. * – originally identified as *P. nanus.*

The alignment generated with default parameters was 885 bp in length and contained 35 sequences. Phylogenetic relationships of *P. nanus* within selected *Paratylenchus* are given in Figure 4 Sequence of *P. nanus* from Virginia was identical to that of *P. nanus* type A from California. New sequence of *P. projectus* from North Dakota was identical to those of previously identified as *P. nanus* type B from South Africa (Van den Berg et al., 2014) or as *P. nanus* from North Dakota (Upadhaya et al., 2019a, b) and South Korea (Mwamula et al., 2020), and now all considered as *P. projectus*.

#### COI *mtDNA* gene

**Figure 5:**
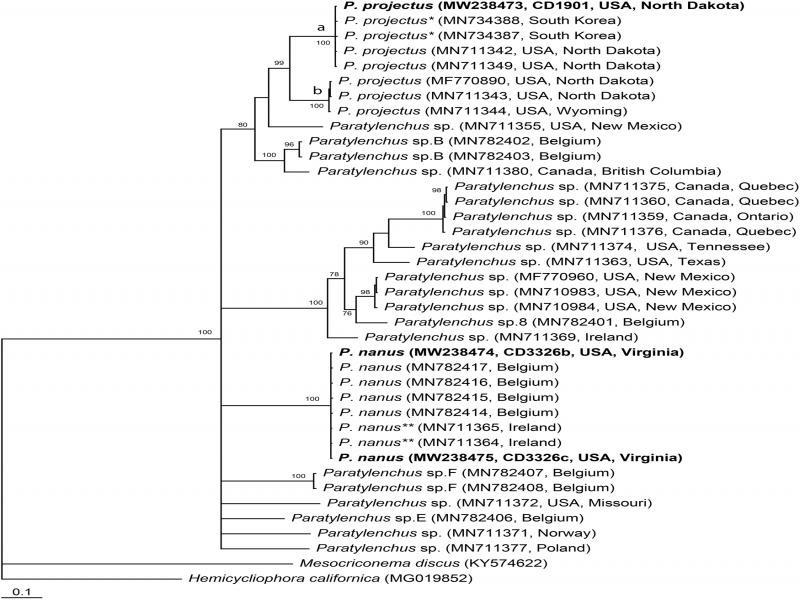
Phylogenetic relationships of *Paratylenchus nanus* with other related species: Bayesian 50% majority rule consensus tree from two runs as inferred from analysis of the *COI* gene sequence alignment under the GTR + I + G model. Posterior probabilities equal or more than 70% are given for appropriate clades. New sequences are indicated in bold. * – originally identified as *P. nanus*, ** *−* originally identified as *Pratylenchus* sp.

The alignment generated with default parameters was 721 bp in length and contained 39 sequences. Phylogenetic relationships of *P. nanus* within selected *Paratylenchus* are given in Figure 5. Sequences of *P nanus* from Virginia were identical to those from *P. nanus* type A from Belgium provided by Etongwe et al. (2020). *Paratylenchus projectus* formed two clades: a and b in the phylogenetic trees. New sequence of *P. projectus* from North Dakota was identical to those of this species from this state by Powers et al. (2020) and those previously identified as *P. nanus* type B from South Korea (Mwamula et al., 2020) and now considered *P. projectus.*

In this study, we consider Four Mile Run, Fall Church, Virginia, USA as the type locality for *P. nanus.* Specimens of *Paratylenchus* collected from this location belong to *P. nanus* molecular type A according to Van den Berg et al. (2014) and, thus, these nematodes should be considered as true representatives of *P. nanus. Paratylenchus* specimens collected from the roots of grasses collected in Devils Lake, North Dakota, in the location also mentioned in Cobb’s description of *P. nanus* are considered here as representatives of *P*. *projectus* as they have been already identified by Raski (1975).

## References

[ref001] Cobb, N. A. 1923. Notes on *Paratylenchus*, a genus of nemas. Journal of the Washington Academy of Science 13:251–257.

[ref002] Etongwe, C. M. , Singh, P. R. , Bert, W. and Subbotin, S. A. 2020. Molecular characterisation of some plant-parasitic nematodes (Nematoda: Tylenchida) from Belgium. Russian Journal of Nematology 28:1–28.

[ref003] Geraert, E. 1965. The genus *Paratylenchus* . Nematologica 11:301–334.

[ref004] Jenkins, W. R. 1964. A rapid centrifugal flotation technique for separating nematodes from soil. Plant Disease Reporter 48:692.

[ref005] Munawar, M. , Powers, T. O. , Tian, Z. L. , Harris, T. S. , Higgins, R. and Zheng, J. W. 2018. Description and distribution of three criconematid nematodes from Hangzhou, Zhejiang province, China. Journal of Nematology 50:183–206, doi: 10.21307/jofnem-2018-010. PMC6909330 30451437

[ref006] Mwamula, O. A. , Kabir, F. Md. , Lee, G. , Choi, I. H. , Kim, Y. H. , Bae, E. -J. and Lee, D. W. 2020. Morphological characterisation and molecular phylogeny of several species of Criconematina (Nematoda: Tylenchida) associated with turfgrass in Korea, as inferred from ribosomal and mitochondrial DNA. Nematology, in press, doi: 10.1163/15685411-bja10003.

[ref008] Powers, T. O. , Harris, T. S. , Higgins, R. S. , Mullin, P. G. and Powers, K. S. 2020. Nematode biodiversity assessments need vouchered databases: a BOLD reference library for plant-parasitic nematodes in the superfamily Criconematoidea. Genome 1–10, doi: 10.1139/gen-2019-0196. 32526150

[ref007] Powers, T. O. , Bernard, E. C. , Harris, T. , Higgins, R. , Olson, M. , Lodema, M. , Mullin, P. , Sutton, L. and Powers, K. S. 2014. *COI* haplotype groups in *Mesocriconema* (Nematoda: Criconematidae) and their morphospecies associations. Zootaxa 3827:101–146, doi: 10.11646/zootaxa.3827.2.1. 25081151

[ref009] Raski, D. J. 1975. Revision of the genus *Paratylenchus* Micoletzky, 1922, and descriptions of new species. Part II of 3 parts. Journal of Nematology 7:274–295. 19308171 PMC2620108

[ref010] Subbotin, S. A. , Sturhan, D. , Chizhov, V. N. , Vovlas, N. and Baldwin, J. G. 2006. Phylogenetic analysis of Tylenchida Thorne, 1949 as inferred from D2 and D3 expansion fragments of the 28S rRNA gene sequences. Nematology 8:455–474.

[ref011] Tanha Maafi, Z. , Subbotin, S. A. and Moens, M. 2003. Molecular identification of cyst-forming nematodes (Heteroderidae) from Iran and a phylogeny based on the ITS sequences of rDNA. Nematology 5:99–111.

[ref012] Tarjan, A. C. 1960. A review of the genus *Paratylenchus* Micoletzky, 1922 (Paratylenchinae: Nematoda) with a description of two new species. Annals of the New York Academy of Sciences 84:329–390.

[ref013] Thorne, G. and Smolik, J. D. 1971. The identity of *Paratylenchus nanus* Cobb, 1923. Proceedings of the Helminthological Society of Washington 38:90–92.

[ref014] Upadhaya, A. , Yan, G. P. and Pasche, J. 2019a. Reproduction ability and growth effect of pin nematode, *Paratylenchus nanus*, with selected field pea cultivars. Plant Disease 103:2520–2526. 31432776 10.1094/PDIS-12-18-2136-RE

[ref015] Upadhaya, A. , Yan, G. P. , Pasche, J. and Kalil, A. 2019b. Occurrence and distribution of vermiform plant-parasitic nematodes and the relationship with soil factors in field pea (*Pisum sativum*) in North Dakota, USA. Nematology 21:445–457.

[ref016] Van den Berg, E. , Tiedt, L. R. and Subbotin, S. A. 2014. Morphological and molecular characterisation of several *Paratylenchus* Micoletzky, 1922 (Tylenchida: Paratylenchidae) species from South Africa and USA, together with some taxonomic notes. Nematology 16:323–358.

